# Genetic variability of attachment (G) and Fusion (F) protein genes of human metapneumovirus strains circulating during 2006-2009 in Kolkata, Eastern India

**DOI:** 10.1186/1743-422X-8-67

**Published:** 2011-02-12

**Authors:** Anurodh S Agrawal, Tapasi Roy, Swati Ghosh, Mamta Chawla-Sarkar

**Affiliations:** 1 Division of Virology, National Institute of Cholera and Enteric Diseases, P-33, C.I.T. Road Scheme XM, Beliaghata, Kolkata-700010, India

## Abstract

**Background:**

Human metapneumovirus (hMPV) is associated with the acute respiratory tract infection (ARTI) in all the age groups. However, there is limited information on prevalence and genetic diversity of human metapneumovirus (hMPV) strains circulating in India.

**Objective:**

To study prevalence and genomic diversity of hMPV strains among ARTI patients reporting in outpatient departments of hospitals in Kolkata, Eastern India.

**Methods:**

Nasal and/or throat swabs from 2309 patients during January 2006 to December 2009, were screened for the presence of hMPV by RT-PCR of nucleocapsid (N) gene. The G and F genes of representative hMPV positive samples were sequenced.

**Results:**

118 of 2309 (5.11%) clinical samples were positive for hMPV. The majority (≈80%) of the positive cases were detected during July−November all through the study period. Genetic analysis revealed that 77% strains belong to A2 subgroup whereas rest clustered in B1 subgroup. G sequences showed higher diversity at the nucleotide and amino acid level. In contrast, less than 10% variation was observed in F gene of representative strains of all four years. Sequence analysis also revealed changes in the position of stop codon in G protein, which resulted in variable length (217-231 aa) polypeptides.

**Conclusion:**

The study suggests that approximately 5% of ARTI in the region were caused by hMPV. This is the first report on the genetic variability of G and F gene of hMPV strains from India which clearly shows that the G protein of hMPV is continuously evolving. Though the study partially fulfills lacunae of information, further studies from other regions are necessary for better understanding of prevalence, epidemiology and virus evolution in Indian subcontinent.

## Background

Acute Respiratory tract infections (ARTI) are a leading cause of morbidity and mortality worldwide [1]. Human metapneumovirus (hMPV), genus Metapneumovirus, family paramyxoviridae first identified in the Netherlands [2], is an important etiological agent of acute respiratory tract infection in almost all age groups. Subsequently it has been identified all over the world [3-6]. Morphologically, hMPV consists of a negative-sense, single stranded and non Segmented RNA that encodes at least 9 distinct proteins [7]. Among them, the two major transmembrame glycoproteins, G and F, stimulate the production of protective immune responses, and therefore, are antigenically significant [8]. F protein promotes fusion of the viral and cell membrane while G protein mediates virus binding to the cell receptor [9].

Genetic analysis on the basis of N, M and F genes have classified hMPV into two distinct groups or genotypes A and B [10-13]. Both genotypes are known to be prevalent throughout the world and circulate in a single season with the switching of predominant group in successive seasons [3,12,14-16]. Unlike the relatively conserved F protein (95% identity at the amino acid level between group A and B), the G protein is highly variable with only 53% amino acid homology between group A and B [17,18].

In developing countries like India, approximately 0.5 million children <5 years of age die due to ARTI [19-21]. We have previously reported prevalence of Influenza A (11±1%), influenza B (5.5± 0.5%) and RSV (7.5 ±1%) among outdoor patients in Kolkata [22,23]. Inspite of its significance as an important respiratory pathogen, there is no information on prevalence and genetic diversity of hMPV strains in India except for one report from Northern India [24]. To partially fulfill this lacuna, the study was done to analyze the extent of genetic variation and the circulation pattern of hMPV in Kolkata during 2006-2009.

## Methods

### Sampling site and Study Population

The study was conducted among patients of all age group exhibiting fever and 2 or more symptoms of ARTI (cold/cough, sore throat, myalagia, body ache) from the outdoor patient ward of hospitals in Kolkata as reported previously [23]. None of these patients were hospitalized. Nasal and/or throat swabs were collected from 2309 patients and were transported in viral transport media (VTM) to the laboratory. The study was approved by the Institutional Ethical Committee and the informed consent was taken from patients or their guardians.

### Extraction of viral RNA

RNA was extracted from 200 ul clinical samples using commercially available RNeasy Mini Kit (Qiagen GmbH, Hilden, Germany) as per manufacturer's instructions.

### Reverse transcription and PCR

For initial screening, amplification of a 416 bp portion of nucleoprotein (N) gene was carried out using primers hmpv1 and hmpv2 by RT-PCR as described earlier [25]. All N positive samples were further amplified by using previously described G and F gene specific primers [12,24]. The resulting PCR products were purified with a Qiagen PCR purification Kit.

### Sequence and sequence analysis

Nucleotide (nt.) sequencing of full length G gene and partial F gene (nt.1-nt.805) was carried out by using ABI Prism Big Dye Terminator v3.1 Cycle Sequencing Ready Reaction Kits in an ABI Prism 3100 Genetic Analyzer (PE Applied Biosystems, Foster City, California, U.S.A) using gene specific forward and reverse primers. Potential N'- and/or O'- glycosylation site/s were predicted by using NetNGlyc 1.0 and NetOGlyc v.3.1 software [26,27]. The multiple and pair wise alignment of deduced amino acid (aa) sequences were performed by using CLUSTAL W software and phylogenetic trees were generated by the neighbor-joining method with the MEGA 5 software as described earlier [28].

### Nucleotide sequence database accession numbers

The hMPV sequences for the 22 G and 8 F genes analyzed in this study have been deposited in GenBank under the accession number HQ599198-HQ599227.

## Results

### Prevalence & age distribution of hMPV

A total of 2309 samples were screened during January 2006 to December 2009 by RT-PCR based amplification of the relatively conserved N gene. Although the age of the patients ranged from 1 month to 50 years, most (≥78%) were below 5 years of age (Table 1). The screening results were confirmed by sequencing and BLAST analysis of N gene amplicon. hMPV was identified in 118 (5.11%) samples in four years. 32 (6.32%) of the 506 samples in 2006, 27 (4.46%) of 605 samples in 2007, 41 (5.84%) of 702 samples in 2008 and 18 (3.63%) of 496 samples in 2009 were positive for hMPV.

**Table 1:** Prevalence of hMPV infection in different age groups among outpatients (n = 2309).

Age Group/ Virus prevalence	0-1 year n = 449	1-2 year n = 782	2-5 yr n = 632	≥ 15 yr n = 446
**hMPV**	4.23%	5.37%	7.28%	2.47%

The hMPV positive samples were found at low frequency (0.5-1%) throughout the year but the majority (≈80%) of the hMPV positive samples were detected during July−November correlating positively with rainfall and high humidity.

### Phylogenetic and antigenic analysis of G protein

**Figure 1:**
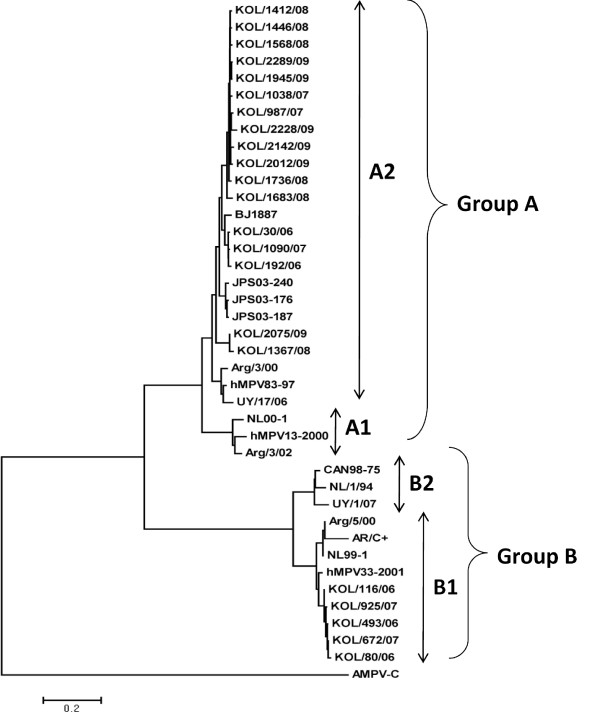
**Phylogenetic analysis of the complete G gene of 22 hMPV Kolkata strains**. Phylogenetic analysis of nt. sequences of hMPV group A and B strains from Kolkata (named with the prefix KOL followed by sample number and the year of collection) with that of other hMPV strains from different subgroups. Trees were built using neighbor-joining algorithm through MEGA 4 program. The tree was rooted with cognate stretch of G gene of strain AMPV-C (GenBank accession number AY198394).

Phylogenetic analysis of 22 Kolkata strains (five representative strains from each year), confirmed two main genetic lineages A and B. Each lineage A and B was further divided into 2 sub-lineage A1 & A2 and B1 & B2 respectively (Figure 1). Interestingly, all the Kolkata strains clustered with A2 and B1 sub-lineage only (Figure 1). During 2006 and 2007, both sub group A2 and B1 co-circulated, with 77% (n = 59) of the circulating strains belonging to A2 subgroup. Of 32 hMPV positive samples in 2006, 26 were as subgroup A2 and 6 as subgroup B1, whereas in 2007, 27 were A2-positive and 8 were B1 positive strains. Interestingly no B1 strains were found in 2008 and 2009 and subgroup A2 remained as dominant strain throughout the study.

Sequence analysis of the complete ORF of G protein, revealed homology ranging from 53.8- 56.4% at nt level and 34.1-35.9% at the aa level between the members of group A and B isolates. Sub group B1 strains shared high percentage of homology with the prototype strain NL/1/99 {91.8%−92.6% (nt.); 89.1%−90.0% (aa)}, whereas subgroup A2 strains revealed homology with the prototype strain CAN97-83 {84.5%−90.8% (nt.); 76%-86.3% (aa)}.

**Figure 2:**
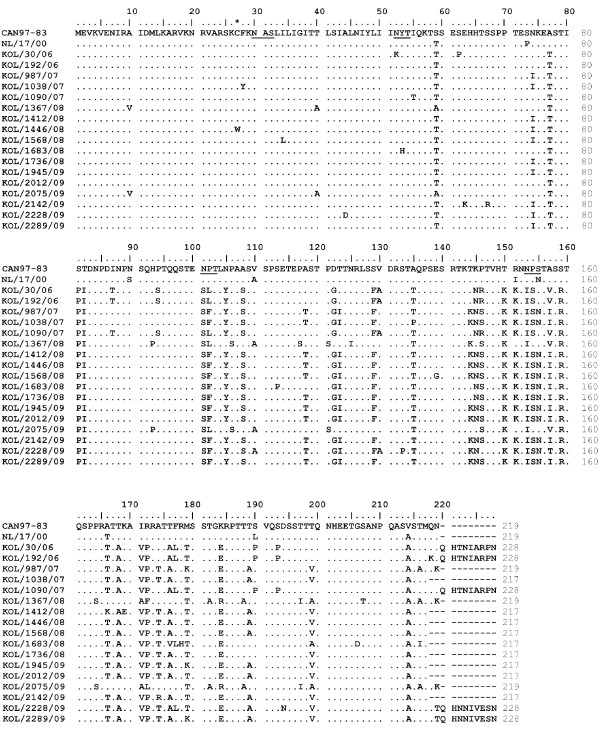
**Deduced amino acid sequence of G ORF of hmpv Group A strains**. Multiple alignment of aa sequences of G protein gene of 17 hMPV subgroup A2 strains from Kolkata. The prototype strain CAN97-83 (GenBank accession number AY485253) were considered as representative for group A. Identical residues are indicated by dots and dashes represent gaps. Cysteine residues are marked with asterisks. Potential N-glycosylation sites are underlined.

**Figure 3:**
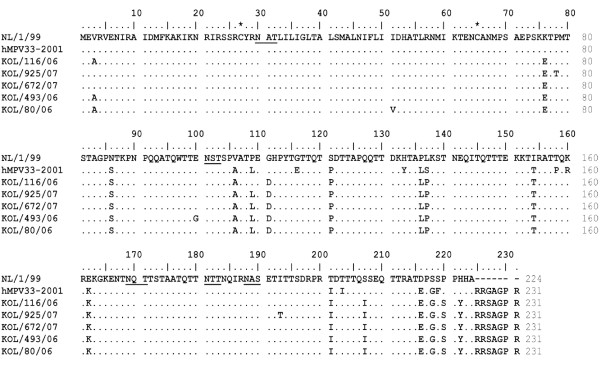
**Multiple alignment of aa sequences of G protein gene of hMPV group B strains**. Deduced aa sequence of complete G ORF of 5 hMPV subgroup B1 strains from Kolkata. The prototype strain NL/1/99 (GenBank accession number AY525843) were considered as representative for group B. Identical residues are indicated by dots and dashes represent gaps. Cysteine residues are marked with asterisks. Potential N-glycosylation sites are underlined.

The alignment of deduced aa sequence of G protein of Kolkata strains with their prototype strains revealed that intracellular and transmembrane regions were highly conserved across the strains (Figure 2 and Figure 3). Most of the aa changes were observed in extracellular domain due to nt substitution and insertion. Changes in position of stop codon have been observed among strains of different subgroups {nt 658 (UAG); nt 652 (UAA), nt 685 (UAG), nt 694 (UGA)}, which correspond to variable lengths in polypeptides. Strains from subgroup A2 used two different stop codons resulting in G proteins of 217aa (UAA), 219aa (UAG) and 228aa (UAG) (Figure 2), whereas subgroup B1 strains terminated at UGA stop codon and exhibited protein of 231 aa in length (Figure 3). For both the subgroup A2 and B1, a cysteine residue at position 27 is strictly conserved among all isolates in the intracellular domain except in one strain Kol/1446/08 which did not contain any cysteine residue. The G ectodomain also has a high content of proline residues, ranging from 7.8% for group B to 10% for group A, which could contribute to an extended, unfolded secondary structure.

The G protein gene sequenced in this study exhibited high content of serine and threonine residues that are potential O-linked sugar acceptors in both subgroups A2 and B1. Serine and threonine content of group A and group B strains was in the range 34.2−37.72% and 29.4−31.18% respectively. The program NetOglyc v. 3.1 predicted 45 to 55 serine and threonine residues to be potentially O-glycosylated with score predictors (G scores) of between 0.5 and 0.8. All the predicted O-glycosylation sites were located in the extracellular region of the subgroup A2 and B1.

The number of N-linked glycosylation site present in the G protein from different subgroup varied from two to six, and only one conserved site (aa 30) at the junction of the intracellular and transmembrane domain [10,29]. The rest of the sites showed subgroup specific conservation: sites 101, 169, 181 & 188 were conserved in all the B1 strains whereas site 52, 145 and 152 was conserved among all the strains of subgroup A2. The predicted N-linked glycosylation sites at aa 52 (subgroup A2) and aa 30 (subgroup B1) exhibited high score of 0.7. Only strain Kol/30/06 lacked the potential site at aa 52 whereas Kol/1367/08 and Kol/2075/09 had lost sites at aa 145 and 152.

### Analysis of the F-gene

**Figure 4:**
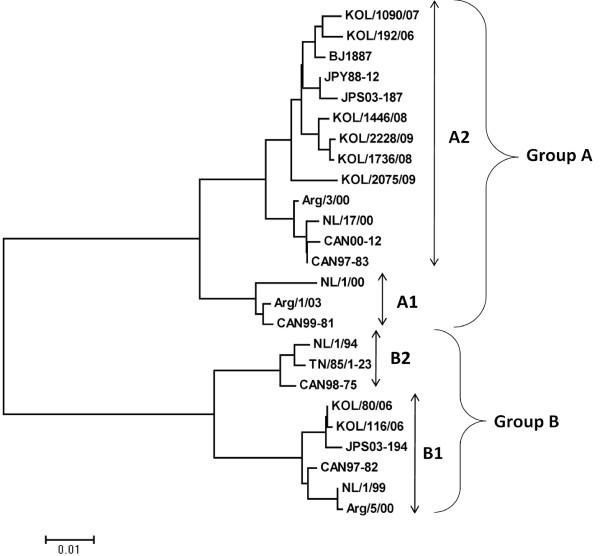
**Phylogenetic analysis of the partial F gene of 8 hMPV Kolkata strains**. Phylogenetic analysis of nt. sequences of hMPV group A and B strains from with that of other hMPV strains. Trees were built using neighbor-joining algorithm through MEGA 4 program. Strains from different subgroups were included in analysis.

Out of 118 hMPV positive Kolkata strains, F-gene was partially sequenced from positive patients covering throughout the study period. Blast analysis and sequence alignment revealed very little difference (≥98% homology) among the strains. Thus the phylogenetic analysis of the F-gene fragment was done with only 8 representative Kolkata strains (two strains per year). The Kolkata strains clustered with A2 (six strains) and B1 (two strains) sub-lineage strains NL/17/00 and NL/1/99 respectively (Figure 4). At the nt level, Kolkata strains shared higher percentage of homology with subgroup B1 prototype strain NL/1/99 (98.1%) than the A2 subgroup strain CAN97-83 (96.05−96.8%). Amino acid alignment of eight partial hMPV F gene (295 aa long) was compared with the prototype strains from Canada and the Netherland (see Additional file 1). For both the subgroup cysteine residues were conserved at position 28, 60, 182, 283 and 292 which could be involved in proper folding of F monomer, as been suggested for hRSV [30]. Some of the important aa changes were subgroup specific which differentiated group A from group B (Table 2).

**Table 2:** List of subgroup specific amino acid changes in the representative Kolkata strains, isolated from 2006-2009.

Position/ Subgroup	61	122	135	139	143	167	175	179	185	233	286	296
**A2**	A	V	T	N	K	D	R	K	D	N	V	K
**B1**	T	I	N	G	Q	E	S	R	A	Y	I	N

## Discussion

In developing countries like India, the mortality and morbidity risk due to ARTI can be 30 times higher than in developed countries [31]. In spite of its importance, very few reports on etiology of ARTI cases are available [22-24]. hMPV is an important cause of ARI, which has been found in both healthy and immunocompromised patients [32,33]. The present study (2006-2009) provided vital insights into the epidemiology and genomic diversity of hMPV strains circulating among patients in Kolkata city, eastern India. To our knowledge, this is the first report on genetic diversity of hMPV strains based on G and F gene sequences from India.

To detect hMPV, initially N gene was chosen as it is highly conserved and has been used in previous studies [33,34]. RT-PCR based detection revealed a significant rate (118/2309) of infection among outpatients with ARI. Of 118 positives, two samples had dual infection with RSV though no differences in clinical symptoms were observed. Compared with Influenza A (11±1%) and RSV (7.5 ±1%), an average 5.11% (4.6%- 6.3%) positivity of hMPV was observed among the same study group [22,23]. This is significantly higher compared to reports from Canada (2.3%), England (2.2%) and USA (4.5%) [4,35,36], but is lower compared to frequency reported from the Netherlands (10%), Australia (9.7%) and Chile (5.4%) [2,16,37]. These variations in detection rates could be attributed to factors such as study population, seasonality and methods for detection. Majority of hMPV was detected from July to Nov (monsoon and autumn), which is similar to previous reports [32,38], but contrary to reports from New Delhi, India and temperate countries [6,24,39], where the high incidence was observed in cold season. This is consistent with seasonality of influenza viruses which follow different seasonality between tropical and temperate countries [40].

Due to similarity between RSV and hMPV, we analyzed genetic diversity of both F and G surface glycoproteins because i) they are two major targets for neutralizing and protective immunity in RSV [8], ii) hMPV F gene is the major antigenic determinant and is classified worldwide into the context of genetic lineages [5,35,41], iii) the G protein has been described as the most variable gene product among hMPV like the G protein of RSV [11,18,29].

Phylogenetic analysis based on nt sequences of G and F gene with the representative strains demonstrated the existence of two group (A and B) and two subgroup A2 and B1. The study demonstrated high prevalence of subgroup A2 (77%) than subgroup B1 (23%) infection. Both group A and B viruses co-circulated for year 2006-07 but later group B virus disappeared. Similar to previous studies [11,29], the G gene sequence alignment showed extensive nt (53.8−56.1%) and aa (34.2−35.9%) variation between these two groups. In addition different length of G polypeptide in strains belonging to different subgroup was also observed due to the usage of different stop codon [5,42,43]. Further studies are required to know whether the changes in stop codon are lineage specific and/or associated with the emergence of new evolutionary lineages, as suggested for RSV [44-46]. On the other hand, accumulation of sporadic aa substitutions and presence of additional and/or absence of N-'and/or O'- glycosylation sites in subgroup A2 and B1 strain from Kolkata provided evidences for constant mutation events which could be either critical for evading immune response/s or may confer enhanced stability that favor gradual establishment of certain local strains over others.

## Conclusion

Since there was only one report on genetic heterogeneity of hMPV strains from northern India, it was extremely difficult to assess the current status of heterogeneity of hMPV strains in the country. For assessing prevalence, susceptible age group, and genetic variation, analysis of hMPV was initiated in addition to other respiratory pathogens in Eastern India. Even though the information is not representative for Indian subcontinent, this study provides the much lacking information on prevalence and genomic diversity of hMPVs in Eastern India.

## Abbreviations

aa: Amino acid; ARTI: Acute respiratory tract infection; hMPV: Human metapneumovirus; nt: Nucleotide; RSV: Respiratory Syncytial virus; RT-PCR: Reverse transcriptase-polymerase chain reaction.

## Competing interests

The authors declare that they have no competing interests.

## Authors' contributions

ASA drafted the manuscript and performed phylogenetic analysis. TR performed screening of clinical samples by RT-PCR. SG collected clinical samples and analyzed epidemiological data. MCS conceived the study, provided guidance and editing of manuscript. All authors read and approved the final manuscript.

## Supplementary Material

Additional file 1 **Alignment of the Deduced amino acid sequence of partial F ORF**. Multiple alignment of aa sequences of F protein gene of hMPV strains from Kolkata. The prototype strain CAN97-83 (GenBank accession number AY485253) is displayed as consensus sequence. Identical residues are indicated by dots and dashes represent gaps. Cysteine residues are marked with asterisks. Potential N-glycosylation sites are underlined. Cleavage site is boxed. Click here for file
