# Supplementary material for: Genetic variability of attachment (G) and Fusion (F) protein genes of human metapneumovirus strains circulating during 2006-2009 in Kolkata, Eastern India
Source: Virol J. 2011 Feb 12;8:67. doi: 10.1186/1743-422X-8-67 (PMC3045894; doi:10.1186/1743-422X-8-67)
Supplement: Additional file 1 — Alignment of the Deduced amino acid sequence of partial F ORF. Multiple alignment of aa sequences of F protein gene of hMPV strains from Kolkata. The prototype strain CAN97-83 (GenBank accession number AY485253) is displayed as consensus sequence. Identical residues are indicated by dots and dashes represent gaps. Cysteine residues are marked with asterisks. Potential N-glycosylation sites are underlined. Cleavage site is boxed. [file 1743-422X-8-67-S1.PDF]

```
             23    30        40         50         60         70         80         90        100
             |.|..*.| ....|....| ....|....| ....|....* ....|....| ....|....| ....|....| ....|....| ..
CAN97-83     YLEESCST ITEGYLSVLR TGWYTNVFTL EVGDVENLTC SDGPSLIKTE LDLTKSALRE LKTVSADQLA REEQIENPRQ SR 124
NL/1/99      ........ .......... .......... .......... T......... .......... .......... .......... .. 124
KOL/80/06    ........ .......... .......... .......... T......... .......... .......... .......... .. 124
KOL/192/06   ........ .......... .......... .......... A......... .......... ....A..... .......... .. 124
KOL/116/06   ........ .......... .......... .......... T......... .......... .......... .......... .. 124
KOL/1090/07  ........ .......... .......... .......... A......... .......... .......... .......... .. 124
KOL/1446/08  ........ .......... .......... .......... A......... .......... .......... .......... .. 124
KOL/1736/08  ........ .......... .......... .......... A......... .......... .......... .......... .. 124
KOL/2228/09  ........ .......... .......... .......... A......... .......... .......... .......... .. 124
KOL/2075/09  ........ .......... .......... .......... A......... .......... .......... .......... .. 124

                  110        120        130        140        150        160        170        180
             ..|....| ....|....| ....|....| ....|....| ....|....| ....|....| ....|....| ....|....| .*
CAN97-83     FVLGAIAL GVATAAAVTA GVAIAKTIRL ESEVTAIKNA LKTTNEAVST LGNGVRVLAT AVRELKDFVS KNLTRAINKN KC 204
NL/1/99      ........ .......... .I........ ....N...G. ..Q....... .......... ......E... ....S...R. .. 204
KOL/80/06    ........ .......... .I........ ....N...G. ..Q....... .......... ......E... ....S...R. .. 204
KOL/192/06   ........ ....S..... .......... .......... ..K....... .......... .......... .......... .. 204
KOL/116/06   ........ .......... .I........ ....N...G. ..Q....... .......... ......E... ....S...R. .. 204
KOL/1090/07  ........ .......... .......... .......... ..K....... .......... .......... .......... .. 204
KOL/1446/08  ........ .......... .......... .......... ..K....... .......... .......... .......... .. 204
KOL/1736/08  ........ .......... .......... .......... ..K....... .......... .......... .......... .. 204
KOL/2228/09  ........ .......... .......... .......... ..K....... .......... .......... .......... .. 204
KOL/2075/09  ........ .......... .......... .......... ..K....... .......... .......... .......... .. 204

                  190        200        210        220        230        240        250        260
             ..|....| ....|....| ....|....| ....|....| ....|....| ....|....| ....|....| ....|....| ..
CAN97-83     DIDDLKMA VSFSQFNRRF LNVVRQFSDN AGITPAISLD LMTDAELARA VSNMPTSAGQ IKLMLENRAM VRRKGFGILI GV 284
NL/1/99      ..A..... .......... .......... .......... .......... ..Y....... .......... .......... .. 284
KOL/80/06    ..A..... .......... .......... .......... .......... ..Y....... .......... .......... .. 284
KOL/192/06   ........ .......... .......... .......... .......... .......... .......... .......... .. 284
KOL/116/06   ..A..... .......... .......... .......... .......... ..Y....... .......... .......... .. 284
KOL/1090/07  ........ .......... .......... .......... .......... .......... ......S... .......... .. 284
KOL/1446/08  ........ .......... .......... .......... .......... .......... .......... .......... .. 284
KOL/1736/08  ........ .......... .......... .......... .......... .......... .......... .......... .. 284
KOL/2228/09  ........ .......... .......... .......... .......... .......... .......... .......... .. 284
KOL/2075/09  ........ .......... .......... .......... .......... .......... .......... .......... .. 284

                  270        280        290
             ..|....| ....|....| ..*.|....| .*..|..
CAN97-83     YGSSVIYM VQLPIFGVID TPCWIVKAAP SCSEKKG 319
NL/1/99      ........ .......... .....I.... .....N. 319
KOL/80/06    ........ .......... .....I.... .....N. 319
KOL/192/06   ........ .......... .......... ....... 319
KOL/116/06   ........ .......... ....TI.... .....N. 319
KOL/1090/07  ........ .......... .......... ....... 319
KOL/1446/08  ........ .......... .......... ......V 319
KOL/1736/08  ........ .......... .......... ....... 319
KOL/2228/09  ........ .......... .......... ....... 319
KOL/2075/09  ........ .......... .......... ....... 319
```
